# A Nano-Bismuth–Silicone Polymer Composite as Shielding for X-Rays in Extreme-Environment Applications: Experimental and Simulation Studies

**DOI:** 10.3390/nano16191198

**Published:** 2026-09-22

**Authors:** Seyyed Saeed Vaezzadeh, Mahmudur Rahman, Kalyan Raj Kota, Masoumeh Ozmaeian, Justin Carter, Robert Kelley Bradley, Merlyn Xavier Pulikkathara

**Affiliations:** 1 Department of Mechanical Engineering, Prairie View A&M University, Prairie View, TX 77446, USA; svaezzadeh@pvamu.edu; 2 Department of Physics, Prairie View A&M University, Prairie View, TX 77446, USA; marahman@pvamu.edu; 3 Mary Kay O’Connor Process Safety Center, Texas A&M Engineering Experiment Station, College Station, TX 77843, USA; rajkota@tamu.edu (K.R.K.); carterjus@gmail.com (J.C.); 4 Matter Mind Analytics LLC., Austin, TX 78735, USA; ozmaian83@gmail.com; 5 Department of Industrial and Systems Engineering, Lamar University, Beaumont, TX 77705, USA; rbradley5@lamar.edu

**Keywords:** nano-bismuth, silicone elastomer composite, X-ray shielding, radiation, polydimethylsiloxane (PDMS), Geant4, Monte Carlo simulation, extreme environments

## Abstract

Radiation shielding is an important consideration in the design and protection of spacecraft electronics and equipment, where conventional metallic shields can add considerable weight and are difficult to integrate with compact or non-planar components. Flexible polymer composites containing high-Z materials offer an alternative approach by combining radiation attenuation with the conformability of an elastomeric matrix. In this work, nano-bismuth is incorporated into a silicone elastomer at loadings of 0–30 wt.% to develop a flexible X-ray shielding composite. The shielding performance of the composites is evaluated experimentally under a 160 kV X-ray source at a total dose of 1 Mrad and compared using Geant4 Monte Carlo simulations. The simulation model is then used to investigate a broader range of bismuth concentrations and composite thicknesses and develop an empirical relationship to predict X-ray attenuation. The results show a clear improvement in X-ray attenuation with increasing nano-bismuth content, demonstrating the potential of silicone/nano-Bi composites as shielding materials for extreme-environment applications, including space.

## 1. Introduction

Spacecraft and satellite electronics operate in a radiation environment that is considerably more complex and severe than that encountered in terrestrial applications. Galactic cosmic rays, solar particle events, trapped protons and electrons within the Van Allen belts, and secondary radiation generated through interactions with spacecraft materials all contribute to the radiation field surrounding onboard systems [1,2]. Over time, the resulting total ionizing dose can degrade semiconductor devices, while single-event effects from high-energy charged particles may cause transient upsets, functional degradation, or even permanent damage [1]. X-ray radiation is an additional, comparatively underrecognized concern for flight hardware. In particular, ground-based sources such as radiographic inspection equipment and avionics test facilities can expose flight electronics to substantial X-ray doses during manufacturing, testing, and qualification. The potential severity of such exposure is illustrated by reported total ionizing dose failure thresholds: unhardened microelectronics may be damaged at doses of only a few hundred rad (in Si), whereas radiation-hardened designs can remain functional at doses about 10^7^ rad [3]. As crewed and robotic missions to the Moon, Mars, and more distant destinations become longer and more complex, the need for shielding approaches that provide effective attenuation without imposing excessive mass becomes increasingly important [4]. Aluminum is the material most widely used in spacecraft structures and, as a result, has also become a conventional radiation-shielding material by default. Lead, meanwhile, remains a traditional reference material for X-ray and gamma-ray shielding in terrestrial and medical applications. Neither material is ideally suited to direct integration with spacecraft electronics. Metallic shields are relatively heavy and rigid, making them more difficult to incorporate around compact components or structures with complex geometries [5]. Lead also presents well-established toxicity concerns during fabrication, handling, and disposal [6]. The mass implications of metallic shielding are illustrated by orbital dose modeling. In one low-Earth-orbit shielding analysis, an aluminum-equivalent areal density of approximately 1–2 g/cm^2^ was reported to reduce a simulated dose from about 75 krad to roughly 7.5 krad [5]. Because launch mass remains a major constraint in spacecraft design, substantial research has focused on lightweight shielding materials that provide radiation protection while also offering mechanical compliance, thermal stability, or structural support [6,7,8]. Polymer-based composites are particularly promising in this regard because their filler content and geometry can be adjusted during processing, enabling shielding layers to conform to electronic housings and other non-planar surfaces [7,8].

A variety of functional fillers has consequently been incorporated into polymer matrices to enhance radiation-shielding performance. Boron- and hydrogen-containing materials, including boric acid and borated polyethylene, have been widely investigated for neutron attenuation, leveraging hydrogen moderation followed by capture via ^10^B [9,10]. A systematic NASA computational and experimental study investigated hydrogen/boron/nitrogen shielding architectures, including hydrogenated boron nitride nanotubes, to attenuate galactic cosmic rays, solar energetic particles, and secondary neutrons [11]. Carbon-fiber composites incorporating carbon nanotubes and boron nitride nanoparticles have also been investigated for simultaneous attenuation of gamma rays and neutrons, while maintaining mechanical properties suitable for structural applications [12]. Similarly, boric-acid-cross-linked poly(vinyl alcohol) hydrogels have been proposed for radiation protection of astronauts [13]. In a related direction, elastomeric composites containing conductive or high-density fillers have been developed to provide electromagnetic interference shielding and self-healing functionality [14]. Taken together, these studies demonstrate the potential of polymeric and elastomeric systems to provide radiation protection while maintaining characteristics that are difficult to obtain with conventional metallic shields. Most of this research, however, has focused on neutrons, gamma rays, or charged particles, while comparatively little attention has been given to elastomeric materials specifically designed for X-ray attenuation.

Bismuth is an attractive filler for this application because its high atomic number (Z = 83) and relatively high density provide strong photon attenuation, with photoelectric absorption contributing substantially in the lower-energy X-ray regime [15]. Compared with lead, bismuth is substantially less toxic and has a more favorable environmental profile, which can be advantageous in applications where material handling and disposal are important considerations [6]. Its potential has already been demonstrated in related composite formats. For example, a barium sulfate/bismuth oxide composite bonded with a silicone-type binder has been reported to provide a shielding rate more than 9% higher than that of a lead sheet of equal thickness, while maintaining good mechanical properties. Composite electronic enclosures incorporating high-Z fillers in place of solid aluminum have likewise been reported to achieve mass savings on the order of 30% [6]. More recent work has begun to examine bismuth/PDMS systems directly. Bismuth nanoparticles bound to cellulose nanofibers within a PDMS matrix have been shown to provide several-fold greater X-ray attenuation per unit mass than bismuth microparticles at equivalent loading [16]. In another study, a five-layer silicone/bismuth oxide/multi-walled carbon nanotube laminate achieved nearly 90% attenuation of scattered X-rays at 60 keV and exhibited improved mechanical strength [17]. A recent investigation of soft wearable shielding materials, however, has pointed out that Bi compound/polymer systems remain limited by dilution from the polymer matrix and by the relatively low atomic number of the non-bismuth constituents present in compound fillers such as Bi_2_O_3_ [18]. To our knowledge, prior studies have not systematically examined high loadings (up to 30 wt.%) of elemental nano-bismuth in a silicone elastomer or evaluated its shielding performance at megarad dose levels relevant to spacecraft electronics, with attenuation predictions validated against Geant4-based Monte Carlo modeling. Silicone- and polymer-based matrices are also attractive for shielding composites because they offer convenient processing, flexibility, and the ability to conform to spacecraft electronic components and housings [7,8]. At the same time, the polymer matrix introduces an important durability consideration, since prolonged radiation exposure can modify the polymer structure and consequently affect mechanical and optical properties, as demonstrated for proton irradiated aluminized Teflon FEP films used in spacecraft thermal-control applications [19].

Experimental characterization alone cannot realistically encompass the broad range of filler concentrations and shield geometries relevant to shielding design. Radiation-transport modeling therefore provides an important complement to experimental measurements. Monte Carlo techniques are particularly useful for this purpose because they explicitly account for the probabilistic interactions of photons and secondary particles within the shielding material. Geant4 [20] has been widely used for photon and particle transport, and its underlying Monte Carlo approach is shared by other established transport codes, including MCNPX [21]. Agreement between different Monte Carlo codes and experimental measurements is not necessarily uniform across all conditions. Benchmarking studies in other transport regimes have shown that the degree of agreement between Geant4, MCNPX, and experimental data can vary with particle type, energy, and absorber thickness [22]. Experimental measurements are therefore essential for establishing confidence in Monte Carlo predictions rather than relying on simulation results alone, which is the validation approach adopted in the present study.

In the present study, nano-bismuth–silicone elastomer composites containing 0–30 wt.% bismuth were fabricated and characterized as candidate X-ray shielding materials for spacecraft electronics. Their attenuation performance was experimentally evaluated after exposure to 1 Mrad of X-ray radiation on glass, as a rough dosimeter [23] and subsequently compared with predictions obtained from Geant4-based Monte Carlo simulations. The simulations were further used to extend the composition and thickness ranges beyond those examined experimentally and to establish an empirical attenuation relationship for nano-Bi/PDMS composites. By combining experimental characterization with radiation-transport modeling, this study provides a framework for assessing bismuth-filled silicone composites as potential alternatives to conventional metallic X-ray shielding materials for space applications.

## 2. Materials and Methods

### 2.1. Materials

Silicone elastomer nanocomposites were prepared using a platinum-catalyzed addition-curing polydimethylsiloxane (PDMS) system consisting of three components: a vinyl-terminated PDMS base polymer (DMS-V31, 1000 cSt, Gelest Inc., Morrisville, PA, USA), a methylhydrosiloxane–dimethylsiloxane copolymer crosslinking agent (HMS-301, 25–35 cSt, Gelest Inc., Morrisville, PA, USA), and a platinum–divinyltetramethyldisiloxane catalyst (SIP6830.3, Gelest Inc., Morrisville, PA, USA). Nano-bismuth (n-Bi) particles were as follows: US1053, 80 nm diameter, and 99.9% purity (US Research Nanomaterials, Inc., Houston, TX, USA).

### 2.2. Composite Fabrication Procedure

These components were incorporated into the silicone matrix at loadings of 0, 5, 15, and 30 wt.% to produce a series of radiation-shielding elastomeric composites. For each batch, 170.5 ± 0.3 g of the vinyl-terminated PDMS base polymer was weighed into a beaker. The crosslinking agent (6.8 ± 0.2 g, corresponding to approximately 3.9 wt.% relative to the base polymer) and the required mass of n-Bi particles were then added. Mixture viscosity was measured at room temperature using a No. 5 Zahn cup (44 mL, stainless steel, orifice diameter 5.28 mm, BAOSHISHAN, Zhengzhou, China), following the ASTM D4212 test procedure [24] to ensure batch-to-batch consistency. Three efflux-time measurements were performed for each batch, with measured efflux times ranging from 33.52 to 35.70 s. Kinematic viscosity was calculated using the manufacturer-supplied calibration relationship, ν = K(t − c), where K = 23 and c = 0 for the No. 5 cup, giving ν = 23t, with t as the efflux time in seconds and ν as the kinematic viscosity in cSt. The resulting viscosity values ranged from approximately 771 to 821 cSt (≈796 cSt on average).

Subsequently, 170 μL of catalyst was added using a micropipette. The components were manually mixed for approximately 1 min to obtain an initial homogeneous dispersion. To further minimize particle agglomeration and improve microstructural homogeneity, the mixture was processed using a three-roll mill (T50-HT, Torrey Hills Technologies, LLC., San Diego, CA, USA) with a front gap of 3 mm, a rear gap of 2 mm, and a front roller speed of 337.5 rpm.

The compounded mixture was then transferred into two-part aluminum molds with cavity dimensions of 14.0 × 18.7 × 0.6 cm and degassed under vacuum (672 Torr; 89.6 kPa) for 6 h, with the molds left uncovered to facilitate the complete removal of entrapped air. Following degassing, the molds were sealed and cured in a convection oven preheated to 180 °C for approximately 15 min. The cured specimens were then demolded and sectioned into smaller specimens (5–10 m^2^) for X-ray irradiation testing. For modeling, 24 specimens were sectioned/stacked to span a range of thicknesses (approximately 2.3–15.5 mm) so that attenuation could be characterized as a function of thickness, not just bismuth loading.

The density and areal density of all four compositions were measured, and the results are presented in Table 1.

To further evaluate the dispersion of Bi nanoparticles at the highest loading, EDS elemental mapping was performed on the 30 wt.% Bi/Si composite. As shown in Figure 1, the Bi signal is distributed throughout the Si matrix, indicating an overall well-distributed Bi phase at the larger spatial scale. However, localized Bi-rich regions are also clearly observed, indicating the presence of agglomeration. This hierarchical distribution suggests that, while some local agglomeration occurs at the microscale, the Bi phase remains distributed across the larger matrix region. Such a distribution is particularly relevant to the shielding performance of the composite, as the overall spatial coverage of the Bi-rich phase can provide effective attenuation pathways while localized agglomeration remains present at smaller length scales.

### 2.3. Attenuation Modeling: Monte Carlo Simulation

Monte Carlo simulations were performed using Geant4 [20] version 11.2.2 to evaluate the X-ray shielding performance of nano-Bi–silicone composites under irradiation conditions consistent with the experimental X-RAD iR-160 system. The simulations quantified photon transmission, attenuation, transmitted dose, and dose reduction factor (DRF) for composites containing 0, 5, 15, and 30 wt.% bismuth.

The simulation geometry consisted of a cubic vacuum world containing a point X-ray source, a rectangular composite shield (10 × 10 × 4 mm), and a downstream scoring volume positioned immediately behind the specimen. The source-to-sample distance was fixed at 70 mm to match the experimental configuration, and geometry overlap checks were performed prior to each simulation. The scoring volume was a thin air slab (G4_AIR, 200 × 200 × 0.1 mm) [20] in direct contact with the rear face of the shield (0 mm gap), 70 mm from the source along the beam axis. The reported quantity was not absorbed dose but an energy-weighted primary-photon transmission: the kinetic energy of each primary photon at its first entry into the scoring volume was summed and divided by the total initial energy of all emitted primaries (10^5^ histories per point). Attenuation was calculated as 100% minus this transmission. Scattered primaries were included at their reduced energy, whereas secondary photons and electrons were excluded. The incident spectrum represented a tungsten-anode X-ray tube operated at 160 kV and 18.75 mA. No filter was used to remove low-energy X-rays, and the anode angle was 10°. A polyenergetic X-ray spectrum was generated numerically over the energy range of 1–159 keV using a simplified tungsten-target bremsstrahlung model. The spectral intensity was calculated according to Z(E_max_ − E)/E, where Z is the atomic number of the target material (Z = 74 for tungsten), E_max_ is the maximum photon energy corresponding to the tube voltage (E_max_ = 160 keV), and E is the photon energy. Characteristic tungsten emission peaks at 55.55 and 66.66 keV were then added with prescribed relative intensities. The resulting spectrum was normalized so that the sum of the energy-bin probabilities was equal to unity and was used as the incident photon-energy distribution in the simulation [25].

The PDMS matrix was modeled using the elemental composition of the repeat unit (C_2_H_6_OSi)n. Composite materials were generated by incorporating nano-bismuth at the selected weight fractions, and the effective densities were calculated using the rule of mixtures. Photon interaction cross-sections were obtained from the Geant4 G4EMLOW database, which is derived from three evaluated data libraries developed at Lawrence Livermore National Laboratory: the Evaluated Photon Data Library (EPDL97) [26], the Evaluated Electron Data Library (EEDL) [27], and the Evaluated Atomic Data Library (EADL) [28]. Electromagnetic interactions were modeled using the FTFP_BERT physics list.

### 2.4. ImageJ 1.54g

Because the glass served as a rough dosimeter, images were acquired and analyzed by assigning an 8-bit grayscale intensity value to each pixel. Each pixel has a value of 0–255: 0 is black and 255 is white. We selected 75 × 75-pixel regions from 6 image regions (unexposed glass, exposed glass, 0% Bi, 5% Bi, 15% Bi, and 30% Bi) and analyzed them in ImageJ to obtain the mean pixel value for each region. To address the quantitative limitation of the ImageJ-based analysis, the glass-darkening results were compared with independent dose-rate measurements obtained under the same X-ray exposure conditions. As shown in Table 2, the attenuation values derived from the glass grayscale analysis showed a strong positive correlation with the attenuation calculated from the dosimeter measurements. Across the 14 experimental data points, the Pearson correlation coefficient was r = 0.925 (R^2^ = 0.856), indicating a strong linear association between the two measurement approaches. Therefore, although the grayscale intensity itself does not directly represent photon fluence or absorbed dose, the independent dosimetry measurements provide experimental support for using glass darkening as a relative/semi-quantitative indicator of X-ray attenuation. The ImageJ-derived values are consequently interpreted as a calibrated relative measure rather than as a direct measurement of photon fluence or absorbed dose.

### 2.5. X-Ray Radiation Setup

The ionizing radiation was generated by a 160 kV, 3000 W X-ray source (X-RAD iR-160, Precision X-Ray, Inc., Madison, CT, USA) to study the effects of total ionizing dose. The X-rays were not hardened as no filter was used to remove the lower energetic X-rays. The dose rate was 24.7 krad/min, the source-to-sample distance (SSD) was 7 cm, and the exposure was timed to accumulate to 1 Mrad. Small squares of silicone composites were placed on a thin glass plate.

## 3. Results and Discussion

The glass exhibited noticeable darkening following X-ray exposure [23]. To quantify the uncertainty associated with the ImageJ-based pixel analysis and to assess the repeatability of the image-processing procedure, an initial validation experiment was conducted using 12 specimens with an identical thickness of 2.90 mm, as shown in Figure 2. All specimens were subjected to X-ray exposure. The specimens were divided into four bismuth-content groups, consisting of three independent specimens for each composition: 0, 5, 15, and 30 wt.% Bi. For each specimen, the pixel intensity within the predefined regions of interest was determined using ImageJ and subsequently converted to the corresponding X-ray attenuation value. The resulting pixel intensities and calculated attenuation averaged values and the standard deviation are presented in Table 3.

The variation observed among specimens with identical composition and thickness confirms the consistency of the ImageJ-based analysis and provides an experimental basis for interpreting differences in attenuation measured in the subsequent thickness-dependent experiments.

Following this repeatability assessment, a second experiment was performed to investigate the effect of specimen thickness on the image-derived attenuation response. The 26 regions corresponding to unexposed glass, exposed glass, and silicone nano-Bi composites containing 0, 5, 15, and 30 wt.% Bi were evaluated within the same test setup. Figure 3 shows the degree of darkening varies with bismuth content, indicating increased X-ray attenuation as Bi loading increases.

### 3.1. ImageJ Results

ImageJ was employed to quantitatively analyze the grayscale intensity within 16 predefined regions of interest (ROIs). The regions corresponded to unexposed glass, exposed glass, and nano-silicone–Bi composites containing 0, 5, 15, and 30 wt.% Bi. The mean grayscale values of the 16 regions are presented in Figure 4. The variation in pixel intensity reflects differences in the X-ray transmission through the tested configurations. Figure 5 presents the corresponding X-ray shielding performance as a function of bismuth loading.

### 3.2. Geant4 Simulation

Each simulation included 50,000 primary photon histories, providing statistical uncertainties below 0.5%. A comparison between the experimental attenuation measurements and the Monte Carlo simulations showed close agreement, supporting the accuracy of the computational model over the four tested compositions at different thicknesses examined experimentally. The agreement between the experimental measurements and Geant4 simulations demonstrates that the simulation framework captures the dominant photon-interaction and attenuation trends of the nano-bismuth–silicone composites over the experimentally investigated range. To provide a comprehensive validation, the comparison in Figure 6 below, was extended across the full experimentally tested thickness range of approximately 2.3–15.5 mm for all four Bi compositions. A total of 25 matched experimental–simulation configurations were evaluated, with the Experimental and simulated results were compared using the total transmitted fraction as the common metric. Across the complete dataset, the simulation showed good agreement with the experimental measurements, yielding an R^2^ of 0.9943, an RMSE of 1.84 percentage points, and a MAPE of 7.29%. These results demonstrate that the Geant4 model reliably reproduces the experimentally observed attenuation behavior across both composition and thickness variations.

### 3.3. Physical Basis for Empirical Model

X-ray transmission follows the Beer–Lambert law [15]:

(1) $$I=I_{0}e^{-\mu \cdot x},$$

where $I_{0}$ and I are the incident and transmitted X-ray intensities, respectively, μ is the linear attenuation coefficient, and x is the material thickness.

For composites, the effective attenuation coefficient depends on composition. For a fixed incident X-ray spectrum, shielding effectiveness is therefore principally determined by composition and thickness [29]:

(2) $$S\left(\%\right)=100\left(1-\frac{I}{I_{0}}\right),$$

where S is the shielding effectiveness (%).

### 3.4. Development of the Empirical Model

Starting from the Beer–Lambert law, the shielding effectiveness of a composite can be expressed as follows:

(3) $$S(\%)=100\left(1-e^{-\mu_{eff}(w)t}\right),$$

where the effective attenuation coefficient is a function of the bismuth loading and sample thickness. In this equation, *w* is the bismuth loading (wt.%), t is the composite thickness (mm), and $\mu_{eff}$ is the composition-dependent effective linear attenuation coefficient, which accounts for the combined photon interaction characteristics of the PDMS matrix and the dispersed bismuth nanoparticles. As the bismuth content increases, $\mu_{eff}$ increases because of the higher probability of photoelectric absorption and Compton scattering associated with the high atomic number (Z=83) and high density of bismuth. Note that $\mu_{eff}$ is also implicitly dependent on the incident X-ray spectrum; the model developed here is therefore valid for the specific source spectrum used in this study.

The empirical model developed in this work represents $\mu_{eff}$ as a function of bismuth loading, allowing shielding effectiveness to be predicted directly from the composite composition. To capture the nonlinear dependence of attenuation on filler concentration observed in the Monte Carlo simulations, $\mu_{eff}$ is represented empirically as:

(4) $$\mu_{eff}\left(w\right)=a_{0}+a_{1}w^{n},$$

where $a_{0}$ represents the attenuation contribution of the neat PDMS matrix, $a_{1}$ quantifies the enhancement produced by the bismuth nanoparticles, and n describes the nonlinear dependence of attenuation on filler concentration. This formulation reduces to the linear rule of mixtures with n=1, which serves as the physical baseline for additive attenuation contributions. Substituting this expression into the Beer–Lambert equation yields the following:

(5) $$S\left(\%\right)=100\left(1-e^{-\left(a_{0}+a_{1}w^{n}\right)t}\right),$$

To account for deviations from ideal exponential attenuation in finite-thickness composite shields, an empirical thickness exponent was introduced, resulting in the following generalized model:

(6) $$S\left(\%\right)=100\left(1-e^{-(a_{0}+a_{1}w^{n})t^{m}}\right),$$

where $a_{0}$, $a_{1}$, n, and m are regression coefficients determined from the simulation data. The exponent n characterizes the influence of bismuth loading and was found to be close to unity (≈0.891, indicating an approximately linear increase in attenuation with increasing nanoparticle concentration. The thickness exponent m was approximately 0.58, suggesting sublinear thickness scaling.

### 3.5. Coefficient Determination by Nonlinear Regression

The empirical coefficients $a_{0}$, $a_{1}$, n, and m were determined using nonlinear least-squares regression applied to all experimental data points as listed in Table 4. The optimization minimized the objective function to the following:

(7) $$\chi^{2}=\sum_{i=1}^{N}\frac{{\left[S_{i}^{pre}-S_{i}^{sim}\right]}^{2}}{\sigma_{i}^{2}},$$

where χ represents the weighted least-squares objective function,${\;S}^{pre}$ and $S^{sim}$ are the predicted and simulated shielding efficiencies, respectively, and $\sigma_{i}$ represents the uncertainty associated with each simulated data point [30].

Parameter estimation was performed in Python 3.11.5 using the *scipy.optimize.curve_fit* function with the Trust-Region Reflective (TRF) algorithm [31]. Model performance was evaluated using the coefficient of determination ($R^{2}$), root-mean-square error (RMSE), and mean absolute percentage error (MAPE) to assess predictive robustness and minimize the risk of overfitting.

The predictive capability of the proposed empirical model was investigated by comparing model predictions with the corresponding experimental results shown in Figure 7.

### 3.6. Goodness-of-Fit and Model Validation

The model exhibited excellent agreement with the Monte Carlo simulation data, achieving an R^2^ of 0.9975, an RMSE of 1.2263%, and a MAPE of 6.2747%. The close agreement between the training and cross-validation results indicates that the model provides accurate and reliable attenuation predictions across the investigated range of nano-bismuth loadings and composite thicknesses.

### 3.7. Extended Simulation Study on Different Configurations

To develop the empirical attenuation model, an extended set of Monte Carlo simulations comprising 1023 configurations was performed. The simulations covered nano-bismuth loadings from 0 to 30 wt.% in 1 wt.% increments and composite thicknesses from 0 to 16 mm in 0.5 mm increments, with the resulting parameter combinations forming the simulation dataset. Each configuration was simulated using 100,000 primary photon histories, resulting in statistical uncertainties below 0.1%.

The predicted attenuation behavior obtained from the resulting empirical model is presented in Figure 8.

The complete simulation dataset is summarized in Figure 9, which illustrates the combined effects of nano-bismuth loading and composite thickness on X-ray attenuation as a heatmap.

## 4. Conclusions

This study demonstrates the potential of nano-bismuth–silicone elastomer composites as X-ray shielding materials for applications in harsh environments such as space and nuclear sites. Silicone composites containing 0–30 wt.% nano-bismuth were tested experimentally under a 160 kV X-ray source up to a total dose of 1 Mrad, and their attenuation was also evaluated independently using Geant4 Monte Carlo simulations. The experimental results showed a consistent increase in attenuation with increasing nano-Bi content. For specimens with a repeated thickness of 2.90 mm, attenuation increased from approximately 15.31% for neat silicone to 27.72%, 42.44%, and 60.39% for composites containing 5, 15, and 30 wt.% nano-Bi, respectively. The grayscale analysis of the X-ray images revealed the same trend, with higher bismuth contents resulting in progressively greater reduction in transmitted X-ray intensity. These single-thickness values were later superseded by a multi-thickness experimental campaign using 24 specimens spanning ~2.3–15.5 mm. The gray scale experiment shows the practical and efficient use of glass to serve as a quantitative ionizing radiation dosimeter for X-rays.

The broader Monte Carlo analysis showed that attenuation was mainly controlled by nano-bismuth concentration and composite thickness. Simulations covering 0–30 wt.% nano-Bi and thicknesses ranging from 0 to 16 mm were used to develop an empirical relationship to estimate attenuation across this design space. The resulting model accounts for the nonlinear effect of bismuth concentration while maintaining a Beer–Lambert-type dependence on attenuation. The fitted exponent of 0.891 for bismuth loading suggests that attenuation increases nearly linearly with nano-Bi concentration over the simulated range, while the thickness exponent of ~0.58 indicates a weaker, sublinear dependence of attenuation on thickness under the investigated X-ray spectrum.

The model also showed a high level of agreement with the simulation results, with an R^2^ of 0.9975, an RMSE of 1.2263%, and a MAPE of 6.2747%. It should be emphasized that the attenuation reported here was obtained under a 160 kV tungsten X-ray spectrum and demonstrates X-ray shielding performance specifically; it does not directly establish shielding performance against the proton, electron, heavy-ion, or secondary-radiation environments encountered in space. The nano-Bi/silicone composite is therefore best regarded as a promising candidate for X-ray/gamma-photon shielding applications, with its relevance to the broader space radiation environment requiring separate validation against those radiation types. Future work will investigate the proton-irradiation shielding performance of these silicone–bismuth composites under terrestrial conditions and compare the results with those of identical materials previously fabricated by our group and deployed on the MISSE-21 sample carrier, where they are currently exposed to the radiation environment of low Earth orbit.

## Figures and Tables

**Figure 1:**
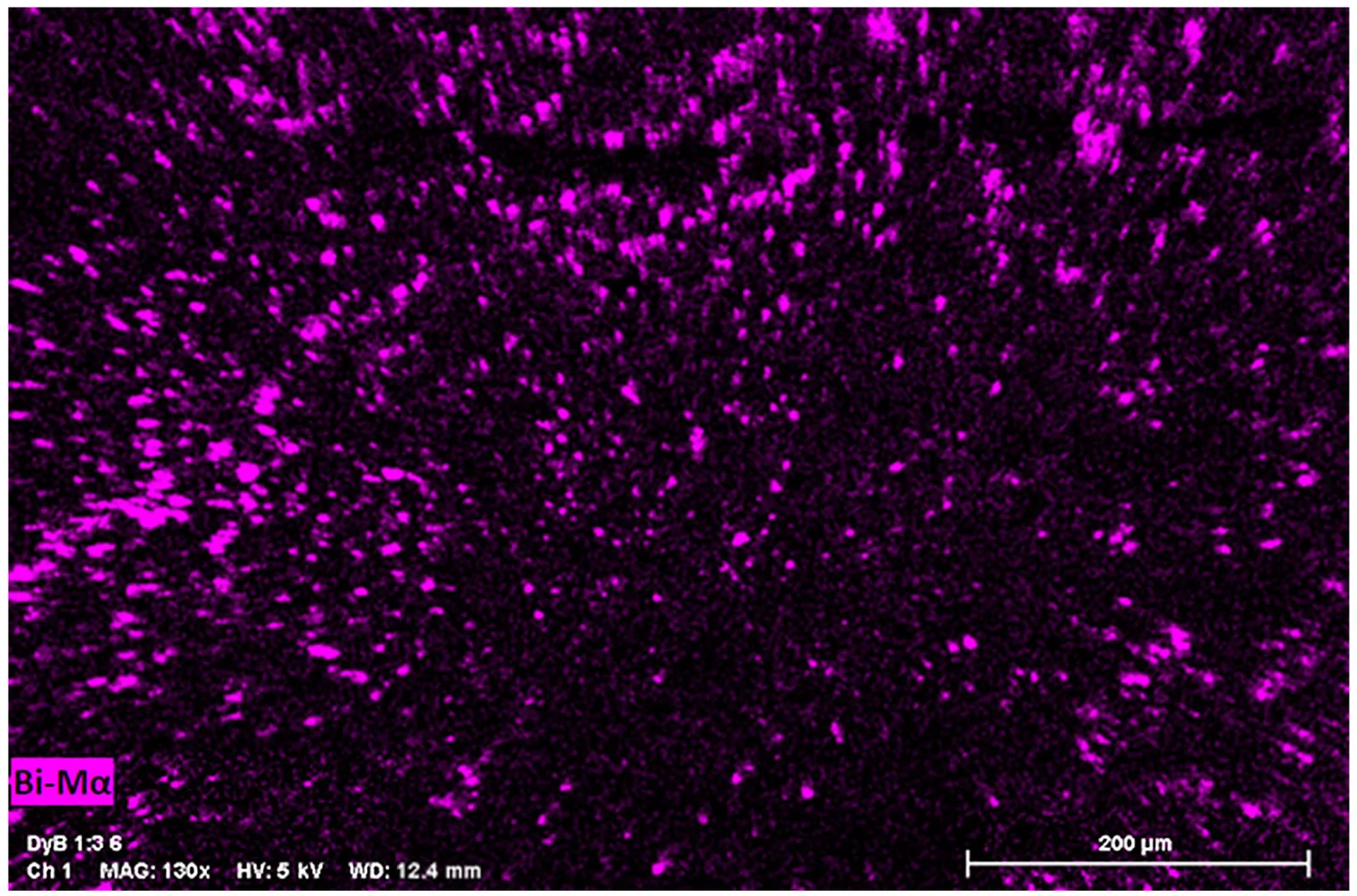
EDS elemental mapping of the 30 wt.% Bi/Si composite, showing the spatial distribution of Bi within the Si matrix. The map reveals an overall distribution of the Bi phase across the examined region, together with localized Bi-rich regions associated with nanoscale agglomeration. The scale bar is 200 μm.

**Figure 2:**
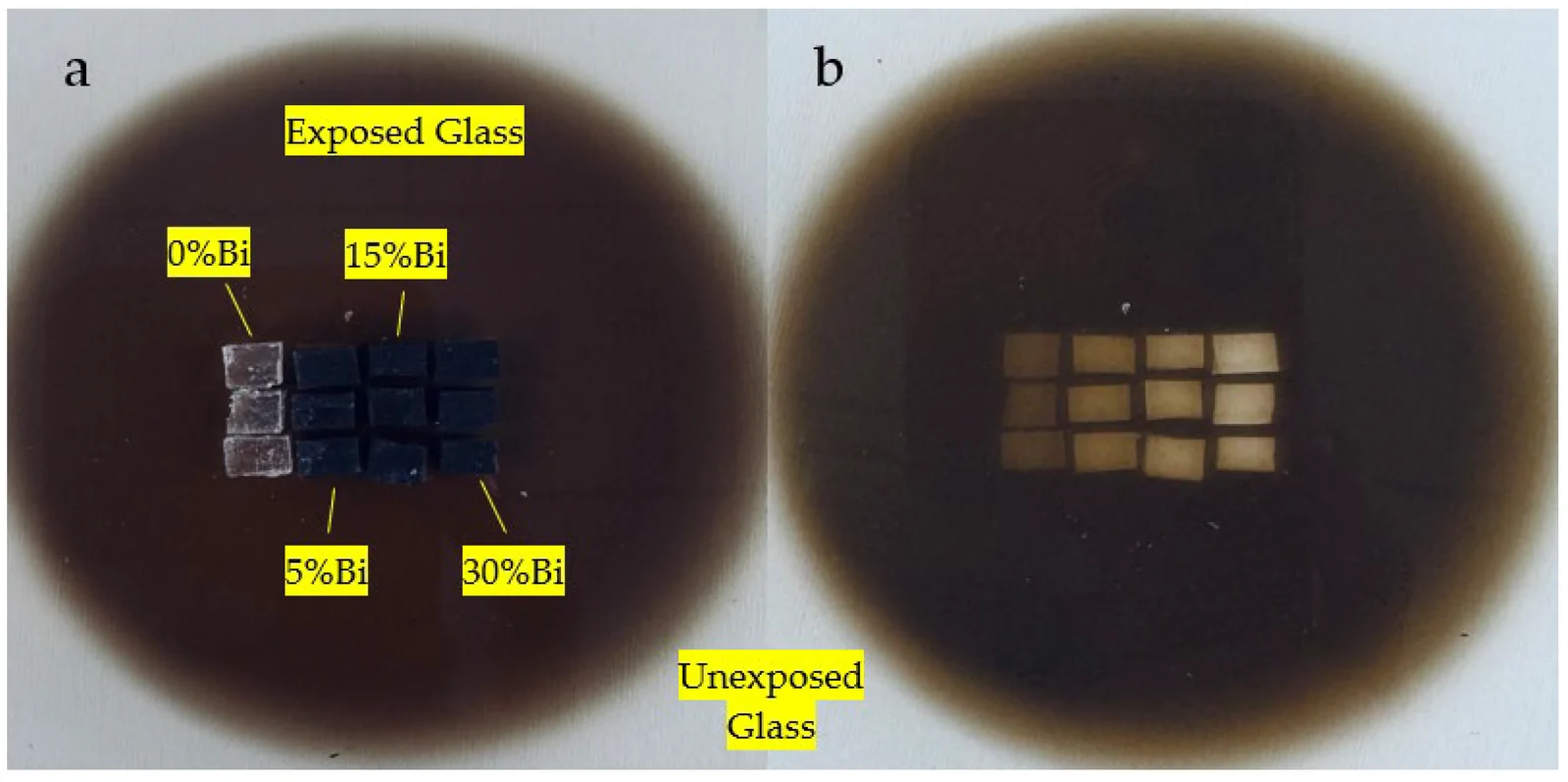
X-ray shielding performance of bismuth-containing samples with varying bismuth contents, demonstrating enhanced shielding with increasing bismuth concentration. (**a**) Samples with 2.90 mm thickness arranged in a square configuration on the glass substrate following exposure to ionizing radiation. The unshielded regions of the glass exhibited visible radiation-induced darkening. (**b**) Glass substrate after removal of the samples, revealing comparatively clear regions beneath the sample locations. The contrast between the shielded and unshielded areas provides visual evidence of the samples’ effectiveness in attenuating the incident ionizing radiation.

**Figure 3:**
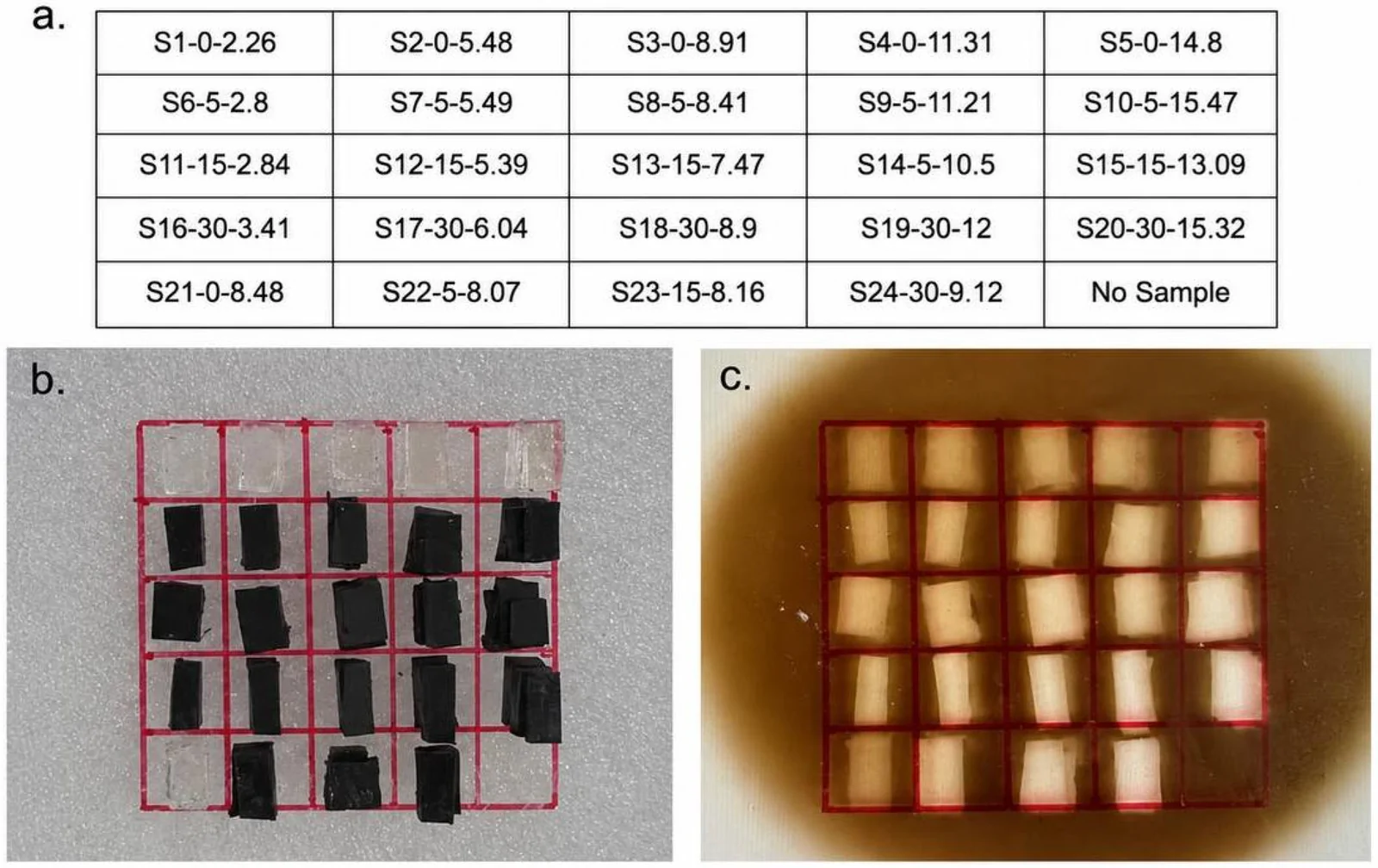
X-ray shielding performance of bismuth-containing samples with varying bismuth content and thickness. (**a**) Sample identification, where each entry indicates the sample number, bismuth content (wt.%), and sample thickness (mm), respectively. (**b**) Arrangement of 24 individual samples with varying bismuth contents and thicknesses on an exposed glass substrate, together with an unshielded glass reference area exposed to the incident X-ray radiation. (**c**) Glass substrate after removal of the samples, showing the radiation-induced changes in glass coloration corresponding to the locations of the samples. The contrast between the exposed and shielded regions provides visual evidence of the effects of bismuth content and sample thickness on X-ray attenuation.

**Figure 4:**
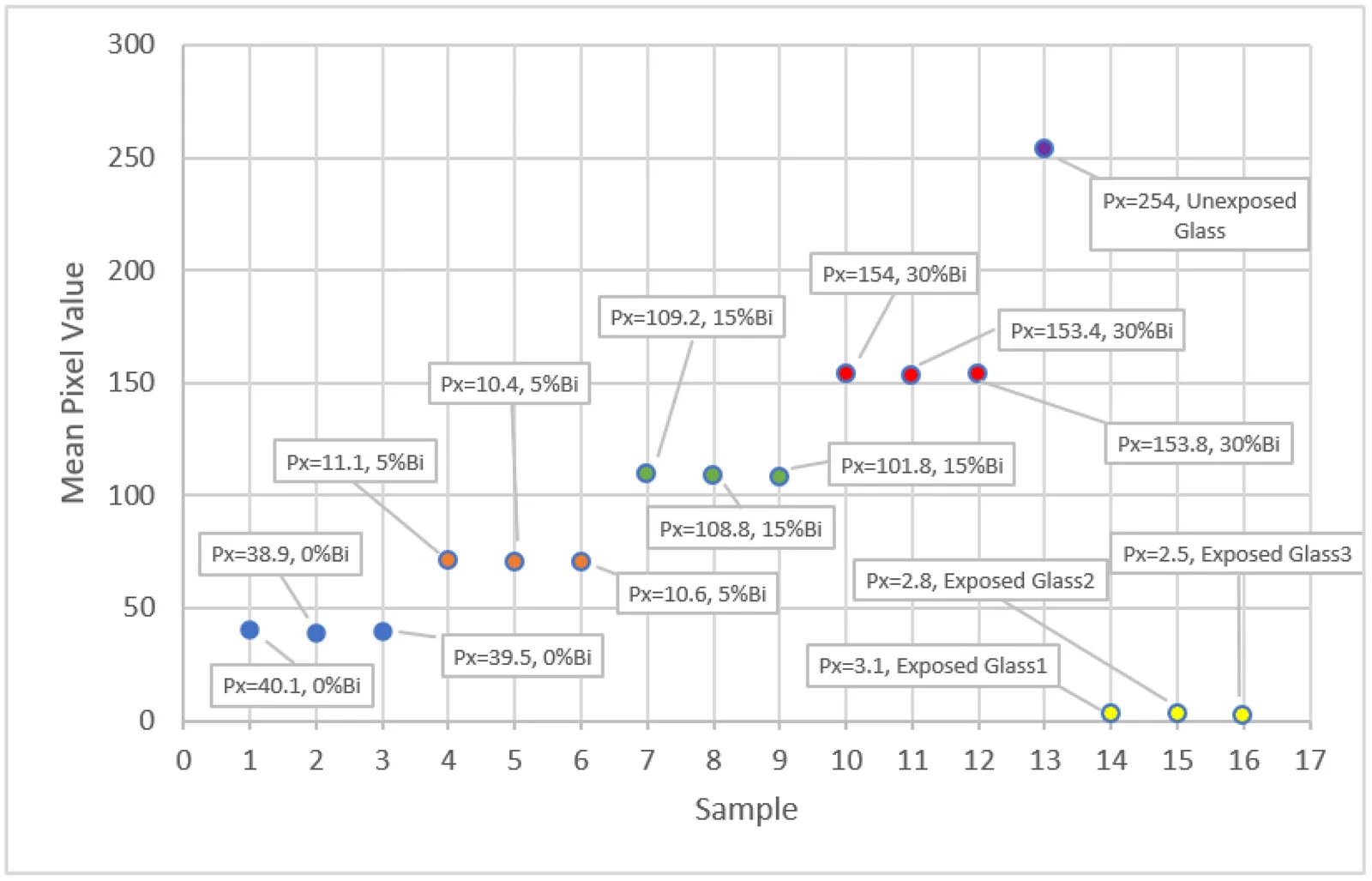
Mean grayscale intensity of the 16 selected regions, including 12 samples and four regions where there were no samples only the glass plate. These data were obtained from ImageJ analysis. The thicknesses of the 12 samples were the same at 2.90 mm.

**Figure 5:**
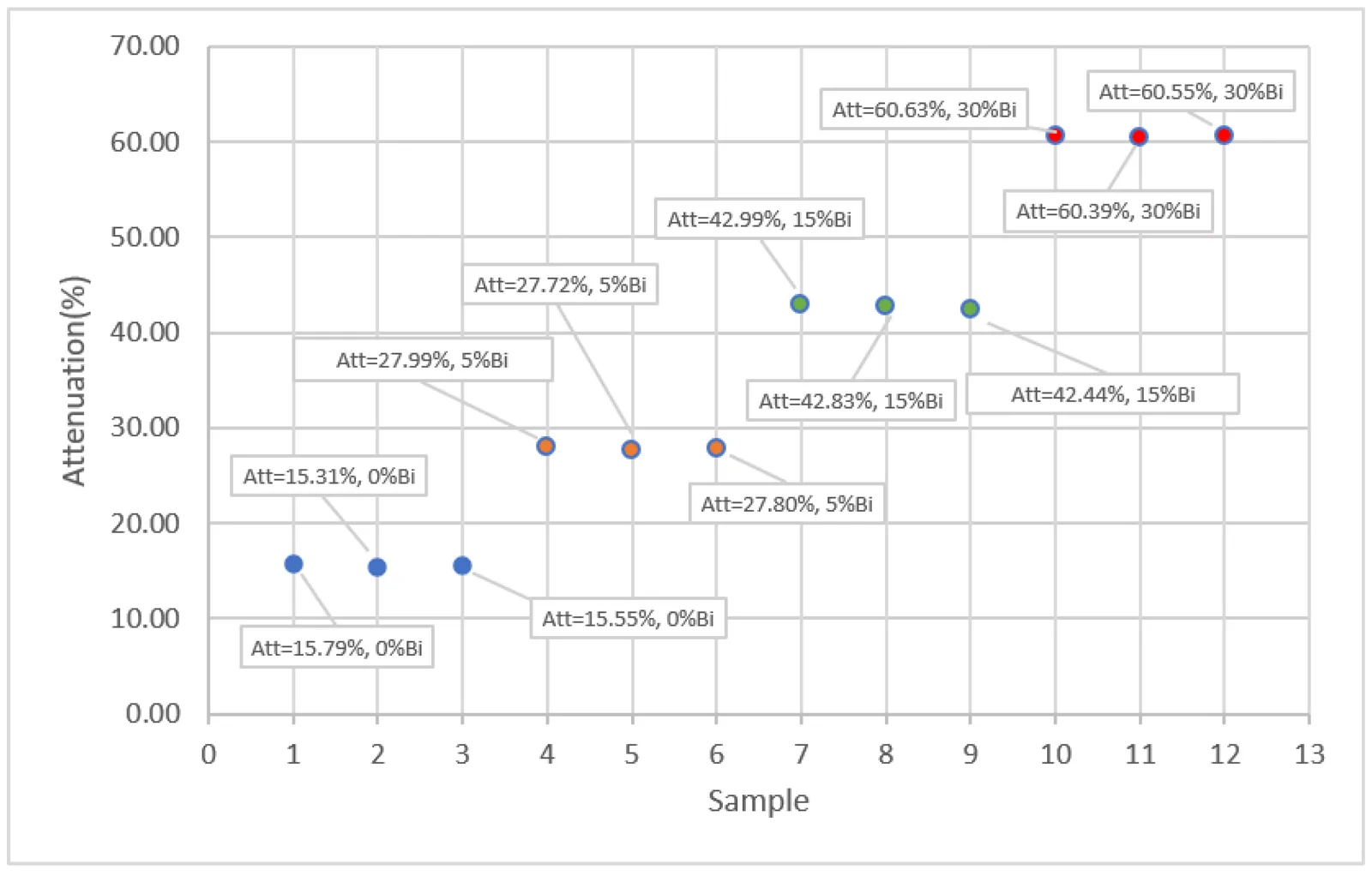
X-ray attenuation performance as a function of bismuth loading, based on the mean grayscale intensity of 12 selected regions analyzed using ImageJ.

**Figure 6:**
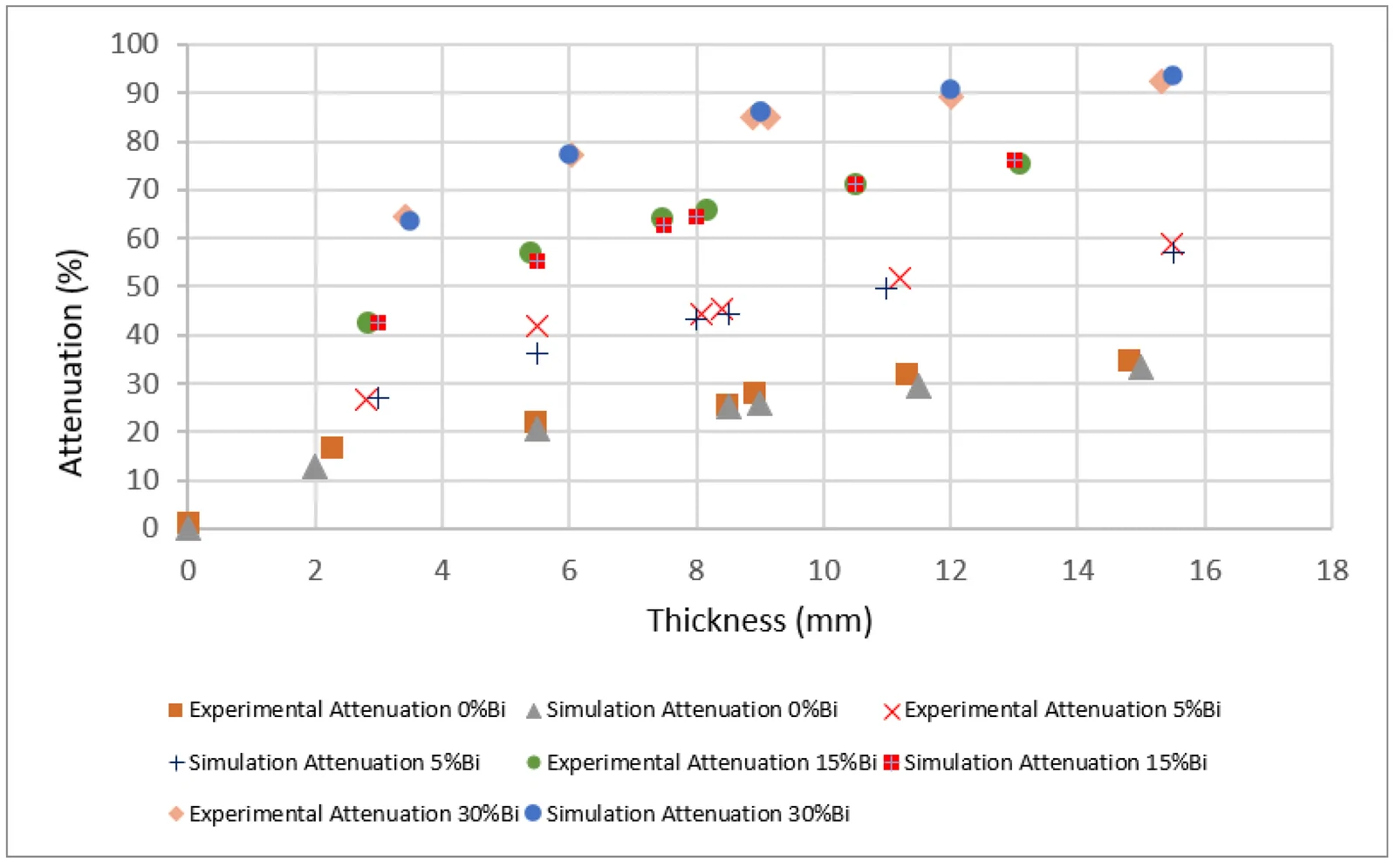
Comparison of the experimental vs. simulation attenuation, with a near perfect fit between the two.

**Figure 7:**
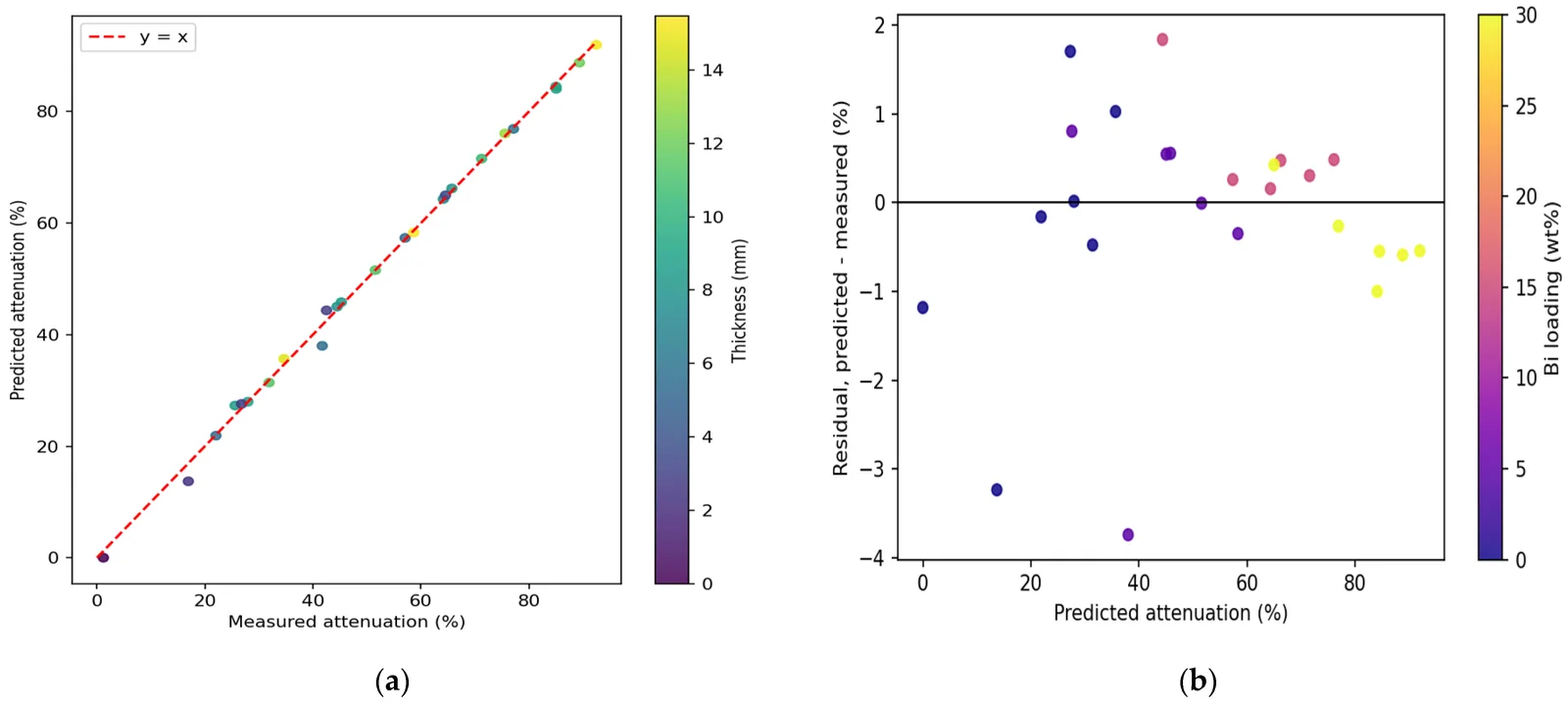
(**a**) Comparison between simulated attenuation values and model predictions for all simulated shielding configurations. The dashed line represents perfect agreement (1:1), demonstrating excellent predictive accuracy. (**b**) Residual analysis of the empirical model. Residuals (predicted − simulated attenuation) are plotted as a function of predicted attenuation, with marker color representing nano-bismuth loading. The residual distribution demonstrates a small, systematic deviation, consistent with the overall fit performance (RMSE = 1.2263% and MAPE = 6.2747%).

**Figure 8:**
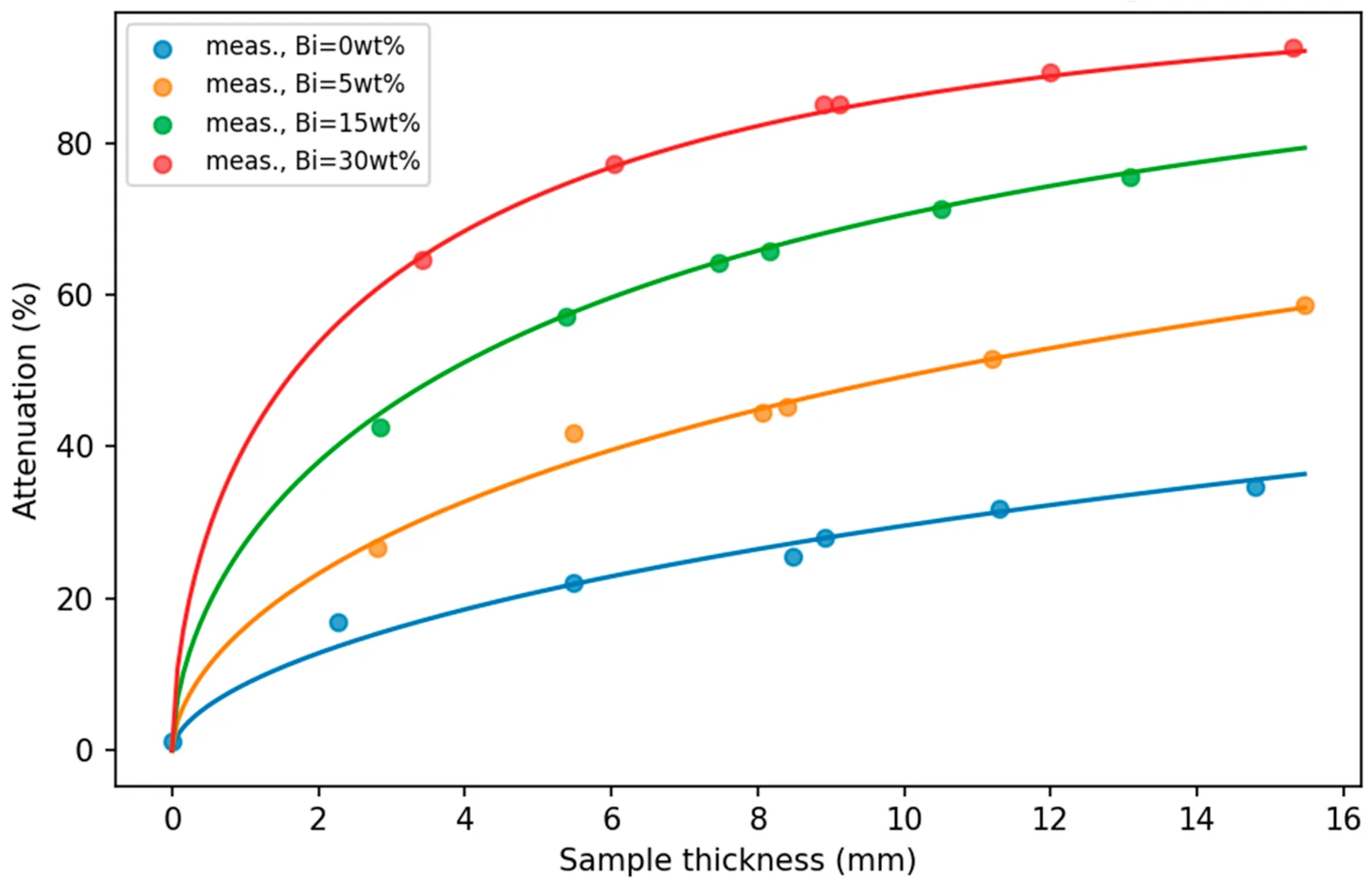
Predicted attenuation behavior obtained from the empirical model for nano-Bi–silicone composites irradiated with a 160 kV tungsten X-ray spectrum. The effect of composite thickness on X-ray attenuation for nano-bismuth loadings.

**Figure 9:**
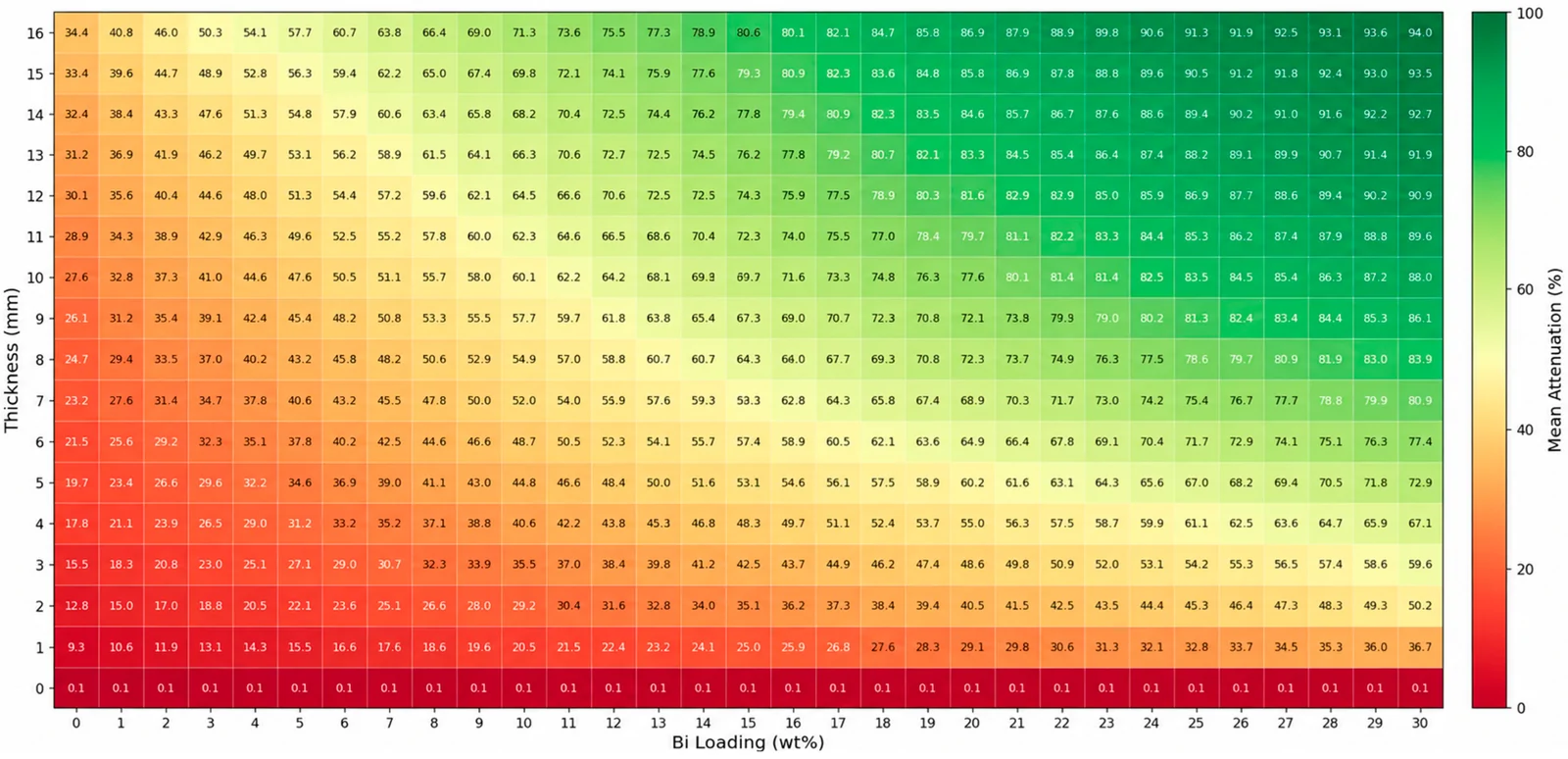
Extended Monte Carlo simulation results illustrating the predicted X-ray attenuation of nano-Bi–silicone composites as a function of nano-bismuth loading (0–30 wt.%) and sample thickness (0–16 mm) under a 160 kV tungsten X-ray spectrum. The heatmap summarizes the 527 simulated shielding configurations used to develop the empirical attenuation model (heatmap and count to be regenerated from the re-run 0–30 wt.% grid).

**Table 1:** Density and areal density of the nano-Bi/silicone composites.

Bi Loading (wt.%)	Density (g/cm^3^)	Areal Density (g/cm^2^)
0	1.02	0.513
5	1.09	0.532
15	1.16	0.571
30	1.41	0.711

**Table 2:** Comparison between ImageJ-derived glass grayscale attenuation values and independent dosimeter measurements across 14 experimental data points (R^2^ = 0.856). These data support the use of glass darkening as a calibrated, semi-quantitative indicator of relative X-ray attenuation.

wt.% Bi	Thickness (mm)	Dose Rate (krad/min)	Dosimeter Attenuation. (%)	Glass Attenuation. (%)	Pixels
0	0	3.552	0	1.18	3
5	2.31	2.724	23.31	16.93	43
30	2.84	1.458	58.95	26.77	68
0	3.22	3.0174	15.05	14.57	37
30	3.28	1.3512	61.96	62.59	159
15	3.96	1.872	47.3	42.52	108
15	5.08	1.665	53.13	57.09	145
5	5.57	2.301	35.22	41.73	106
15	5.61	1.5324	56.86	55.9	142
5	5.87	2.1744	38.78	39.7	101
0	5.89	2.7576	22.36	22.05	56
30	6.03	0.8028	77.4	77.17	196
30	6.11	0.7866	77.85	76.77	195
0	6.18	2.7564	22.4	23.23	59

**Table 3:** Mean grayscale pixel intensity obtained from ImageJ analysis of exposed glass and silicone–Bi composite samples. Values are reported as mean ± standard deviation.

wt.% Bi	Glass	0	5	15	30
Attenuation (%) from Glass	1.10 ± 0.12	15.55 ± 0.24	27.84 ± 0.14	42.75 ± 0.28	60.52 ± 0.12
Pixel	2.80 ± 0.30	39.50 ± 0.60	70.70 ± 0.36	108.67 ± 0.58	153.67 ± 0.58

**Table 4:** Model coefficient.

Coeff	Physical Meaning	Fitted Value ± σ	Units
a_0_	Baseline attenuation	0.091519 ± 0.00446	mm^−m^
a_1_	Bi-loading sensitivity	0.020343 ± 0.001802	mm^−m^·wt.%^−n^
n	Bi-loading exponent	0.891108 ± 0.025706	dimensionless
m	Thickness exponent	0.583703 ± 0.018982	dimensionless

## Data Availability

The raw data supporting the conclusions of this article will be made available by the authors on request.
